# Proline Amide Catalyzes Formation of Toxic Crotonaldehyde from Acetaldehyde Under Physiologically Relevant Conditions

**DOI:** 10.1002/cbic.202500138

**Published:** 2025-05-21

**Authors:** Liam A. Thomas, Vicki L. Emms, Dipti Vashi, Louise Fairall, John W. R. Schwabe, Richard J. Hopkinson

**Affiliations:** ^1^ Institute for Structural and Chemical Biology and School of Chemistry University of Leicester Henry Wellcome Building, Lancaster Road Leicester LE1 7RH UK; ^2^ Institute for Structural and Chemical Biology and Department of Molecular and Cell Biology University of Leicester Henry Wellcome Building, Lancaster Road Leicester LE1 7RH UK

**Keywords:** acetaldehyde, alcoholism, crotonaldehyde, mutation, organocatalysis

## Abstract

Crotonaldehyde is a human toxin that reacts with nucleophilic groups on DNA and proteins. Putative crotonaldehyde‐derived adducts on DNA are reported in cells and patients after ethanol exposure, which implies that crotonaldehyde is formed in cells. Here, we show that proline amide, which is a model of *N*‐terminal proline‐containing proteins, catalyzes the aldol condensation of the ethanol metabolite acetaldehyde to crotonaldehyde under physiologically relevant conditions. This reaction is more efficient at neutral pH than under acidic or basic conditions, but is inhibited by competing imidazolidin‐4‐one formation. Crotonaldehyde formation is also slower than the analogous aldol condensation of propionaldehyde. Comparative studies additionally suggest that proline amide is a more efficient catalyst than other amino acid amides. Overall, the work evidences a biochemically plausible mechanism for intracellular crotonaldehyde formation and implies that proline amide derivatives can catalyze aldol chemistry in humans.

## Graphical abstract

Crotonaldehyde‐derived DNA lesions are reported in patients after ethanol exposure, which implies that crotonaldehyde is produced after ethanol ingestion. Here, we show that proline amide, which mimics *N*‐terminal proline residues on human proteins, can catalyze crotonaldehyde formation from the ethanol metabolite acetaldehyde under physiologically relevant conditions. The work therefore provides a chemically plausible mechanism for crotonaldehyde formation in humans.

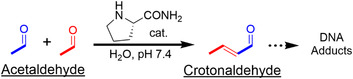

## 1 Introduction

Crotonaldehyde (CrH) is an electrophilic small molecule that is toxic and carcinogenic to humans.^[^ ^1^, ^2^ ^]^ CrH reacts with nucleophilic groups on biomolecules such as sulfur and nitrogen atoms, leading to function‐altering effects.^[^ ^3^, ^4^, ^5^ ^]^ In DNA, adducts derived from electrophiles such as CrH are generally genotoxic; however, the degree of toxicity is likely dependent on the structures of the adducts and their stabilities. It is also possible that electrophiles undergo chemical transformations in cells, leading to new species with different biological functions.

**Figure 1:**
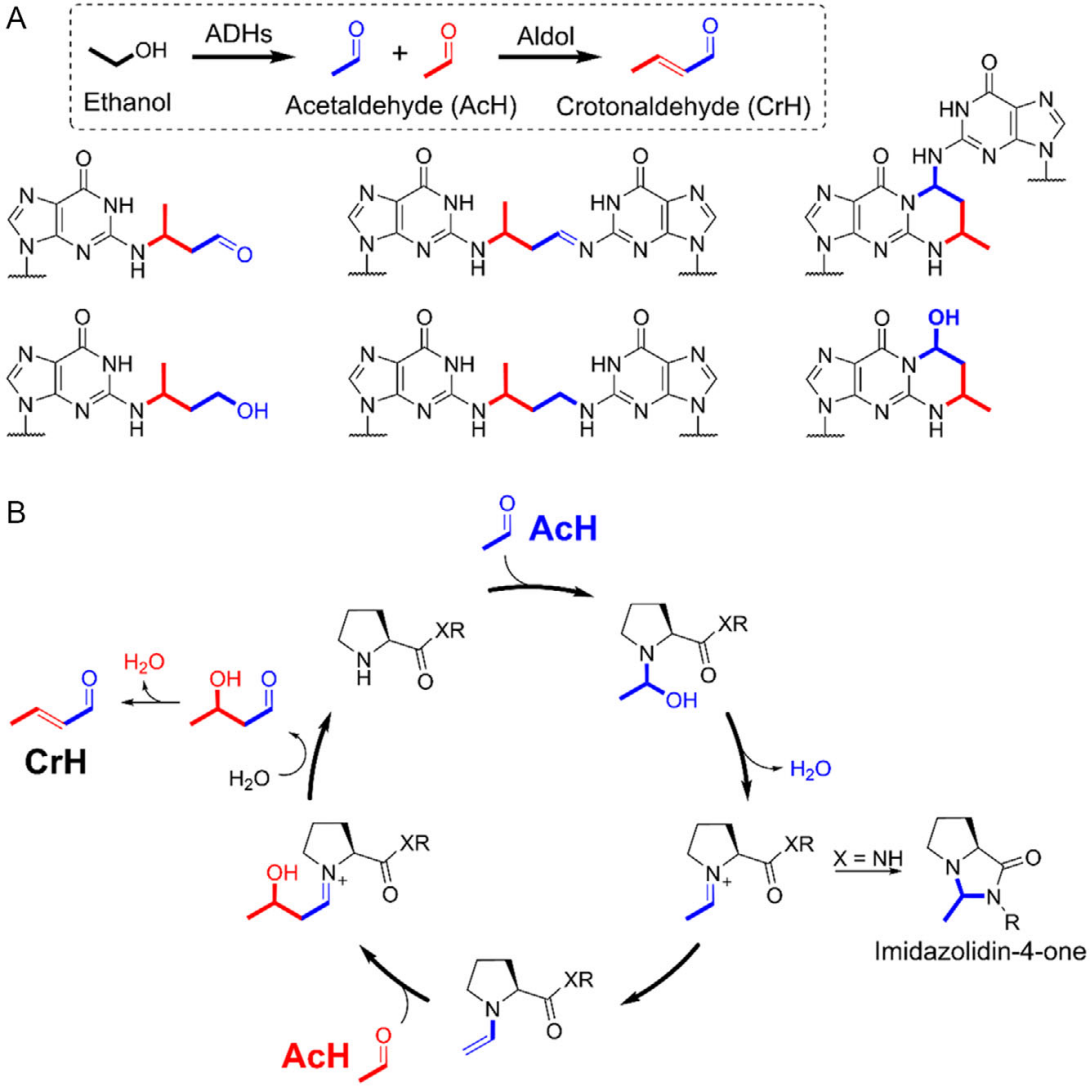
A) Structures of putative CrH‐derived adducts on guanine bases reported on DNA exposed to ethanol or AcH. AcH is produced in cells during oxidative metabolism of ethanol, which is catalyzed predominantly by alcohol dehydrogenases (ADHs). Note, adducts in the bottom left and middle are detected after treatment with a reducing agent. B) Proposed mechanism for proline‐catalyzed formation of CrH from AcH. Proline amide can also form an imidazolidin‐4‐one with aldehydes such as AcH.

As an α,β‐unsaturated aldehyde, CrH is electrophilic at both the aldehyde carbon (C1) and at the β‐alkenyl carbon (C3), with reactions at the alkene producing more stable products.^[^ ^6^ ^]^ Putative CrH‐derived adducts have been reported on DNA‐derived nucleosides from cells and patients exposed to ethanol, which implies that CrH might be formed during ethanol exposure (**Figure** **1**A).^[^ ^1^, ^7^, ^8^, ^9^, ^10^ ^]^

AcH is an intermediate of ethanol metabolism and, like CrH, reacts with guanine bases to induce genotoxicity.^[^ ^6^ ^]^ Aldol condensation of two AcH molecules can lead to the formation of CrH. However, this reaction is likely to be slow under physiologically relevant conditions due to disfavored enol/enolate formation in water. In organic synthesis, aldol chemistry is often accelerated by the addition of an amine catalyst, which can promote the formation of intermediate enamines.^[^ ^11^ ^]^ Proline is commonly used as an enantioselective organocatalyst for aldol reactions in organic solvents but is not routinely used to catalyze aldol chemistry in water (although some reports exist).^[^ ^12^, ^13^, ^14^, ^15^, ^16^, ^17^ ^]^ However, proline amide has been widely reported to catalyze aldol reactions in water,^[^ ^12^ ^]^ while emerging evidence suggests that proteins with *N*‐terminal proline residues (i.e., proline amides) can catalyze aldol reactions in water in certain contexts (Figure 1B).^[^ ^16^, ^17^, ^18^, ^19^ ^]^ *N*‐terminal proline residues are additionally susceptible to inactivating cyclisation reactions with aldehydes, forming imidazolidin‐4‐ones (Figure 1B).^[^ ^17^, ^20^ ^]^

Here, we report NMR studies on the proline amide‐mediated reactions of AcH in buffered water. Time course analyses reveal that proline amide accelerates CrH formation from AcH at pH 7.4, while the reaction efficiency is dependent on pH, AcH concentration, and the efficiency of competing imidazolidin‐4‐one formation. Addition of benzaldehyde to the reaction mixture resulted in comparably efficient formation of cross‐aldol cinnamaldehyde with AcH after 24 hours, while comparative studies with other proteinogenic amino acid amides reveal that proline amide is the most efficient CrH‐forming organocatalyst tested. Overall, our studies therefore confirm proline amide to be an organocatalyst under physiologically relevant conditions and also provide a chemically plausible mechanism for CrH formation following ethanol exposure.

## 2 Results and Discussion

**Figure 2:**
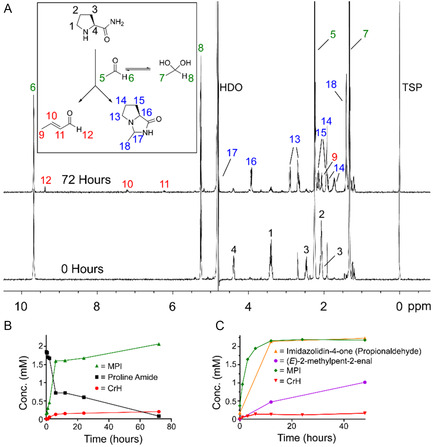
A) ^1^H NMR spectra of a reaction mixture of proline amide with AcH immediately after mixing (bottom) and after 72 h incubation at 25 °C (top). ^1^H resonances corresponding to proline amide, AcH, CrH, and the imidazolidin‐4‐one MPI are highlighted. B) Graph showing time‐dependent formation of CrH and MPI in a mixture of proline amide and AcH at 25 °C. MPI is the major product but CrH grows in concentration during the analysis. C) Graph showing time‐dependent formation of aldol products and imidazolidin‐4‐ones from mixtures of proline amide and either AcH or propionaldehyde at pH 10. Aldol catalysis is more proficient with propionaldehyde.

Initial experiments focused on profiling CrH formation in phosphate‐buffered water using NMR. AcH (20 mM) and proline amide (2 mM) were mixed in 100 mM sodium phosphate solution in 9:1 H_2_O:D_2_O at pH 7.4—analysis by ^1^H NMR was then conducted periodically over 72 h incubation at 25 °C by ^1^H NMR. The spectra revealed time‐dependent formation of new ^1^H resonances that correlated with the formation of two major reaction products (**Figure** **2**A,B). ^1^H resonances corresponding to AcH and proline amide decreased in intensity during the analysis, suggesting that both starting materials are consumed during the reaction. The two new products were assigned as CrH and 3‐methylhexahydro‐1H‐pyrrolo[1,2‐c]imidazol‐1‐one (MPI, an imidazolidin‐4‐one), which is the aminal condensation product of proline amide and AcH (Figure S1–S4, Supporting Information). CrH formation was confirmed by the addition of an authentic standard (Figure S4, Supporting Information). MPI was the most abundant product during the analysis and correlated with the full reaction of proline amide (2 mM after 72 h, Figure 2B). CrH concentrations were much lower than MPI but increased over time and reached a maximum concentration of 200 μM after 72 h (Figure 2B). Other low‐level species were also present in the spectra, although their low abundance precluded full characterization. No unequivocal evidence was observed for the CrH (Z)‐isomer or for 3‐hydroxybutanal, which is presumably an intermediate in the formation of CrH (Figure 1B). We also did not see evidence for a Mannich‐type intermediate (i.e., a 3‐amino derivative)^[^ ^21^, ^22^ ^]^ although it is possible that such species are too transient or low‐level to be detected under the analysis conditions. Importantly, no significant CrH formation was observed in a sample without added proline amide, while resonances corresponding to partially deuterated AcH, which can form during reversible enamine formation in deuterated solvents, were more prominent in the sample with proline amide. Analogous reactivity was observed at 37 °C (Figure S5, Supporting Information). Replacing AcH with propionaldehyde resulted in the formation of 2‐methylpent‐2‐enal (i.e., the analogue of CrH) and the equivalent imidazolidin‐4‐one to MPI (Figure S6–S9, Supporting Information).

Formation of 2‐methylpent‐2‐enal (putatively assigned as the *E* isomer) was more efficient than CrH formation at 25 °C, while formation of the imidazolidin‐4‐one was also faster than MPI formation (Figure S7, Supporting Information).

**Figure 3:**
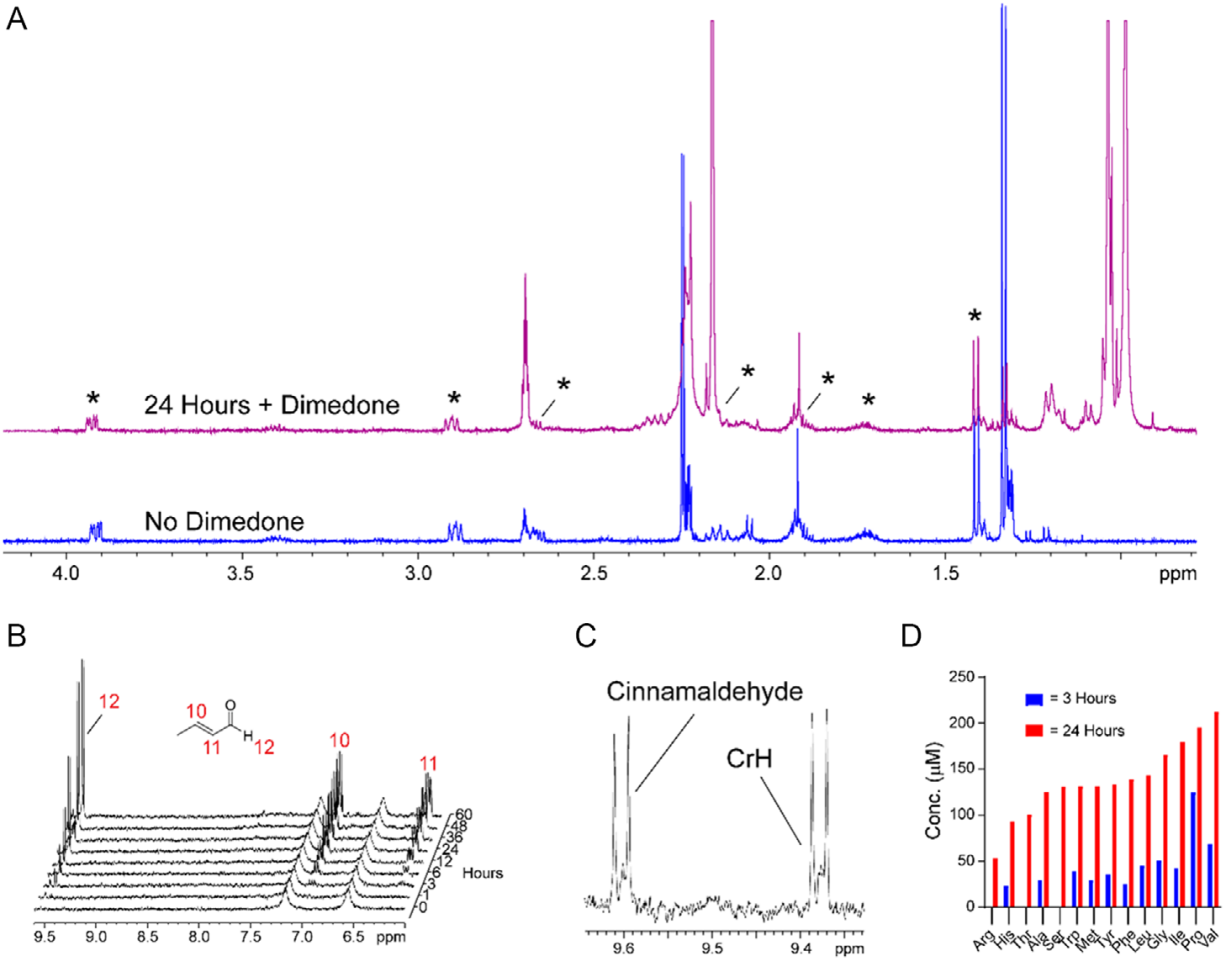
A) ^1^H NMR spectrum showing formation of both CrH and cinnamaldehyde in a mixture of proline amide and AcH and benzaldehyde after 24 h at 37 °C. CrH and cinnamaldehyde concentrations are comparable. B) ^1^H NMR spectra of a mixture of proline amide and AcH after addition of the aldehyde scavenger dimedone. No significant degradation of MPI is observed after the addition of dimedone (24 h after mixing, purple). C) ^1^H NMR spectra showing time‐dependent CrH formation in a mixture of proline amide and AcH (10 equivalents) in acetonitrile. CrH formation is markedly more efficient than in buffered water. No MPI formation is observed. D) Bar graph showing concentrations of CrH in mixtures of AcH and different amino acid amides incubated at 37 °C. Proline amide is the most proficient CrH‐forming organocatalyst after 3 h incubation.

We then conducted the reactions under acidic (pH 5) and alkaline (pH 10) conditions at 25 °C. At pH 5, no reaction with AcH/propionaldehyde and proline amide was observed, which implies that deprotonation events are important for reaction efficiency (as expected for aldol reactions). At pH 10, CrH and (E)‐2‐methylpent‐2‐enal formation was observed in the presence of proline amide; however, CrH formation was less efficient than at pH 7.4, while (*E*)‐2‐methylpent‐2‐enal formation was accelerated and reached a maximum concentration of 1 mM after 48 h (Figure 2C). No significant CrH or (*E*)‐2‐methylpent‐2‐enal formation was observed in the absence of proline amide. Interestingly, imidazolidin‐4‐one formation was more proficient with both AcH and propionaldehyde at pH 10 than at pH 7.4 (no reaction at pH 5). As imidazolidin‐4‐ones are inactive as organocatalysts, these results might suggest that the two imidazolidin‐4‐ones have different stabilities, although both species were stable to treatment with the aldehyde scavenger dimedone at pH 7.5 (**Figure** **3**A, note dimedone is a more efficient scavenger of AcH than of propionaldehyde). Replacing buffered water with acetonitrile precluded MPI formation and induced markedly more efficient formation of CrH (Figure 3B). Therefore, it appears that reducing imidazolidin‐4‐one formation enables more proficient aldol catalysis.

We then tested whether proline amide can catalyze cross‐aldol chemistry under our conditions. For this experiment, we incubated proline amide with equimolar equivalents of AcH and benzaldehyde (each at 10‐fold excess) and monitored the reaction after 24 h at 37 °C. CrH formation was observed alongside formation of another aldol product, which was assigned to cinnamaldehyde by comparison with an authentic standard. CrH and cinnamaldehyde were formed at near‐identical concentrations (30 and 29 μM respectively), which implies that the enamine–aldehyde addition step is not rate‐limiting under the tested conditions (Figure 3C).

CrH formation was then monitored in the presence of different amino acid amides. The concentrations of CrH were compared in samples of AcH incubated with amino acid amides derived from either alanine, arginine, glycine, histidine, isoleucine, leucine, methionine, proline, serine, threonine, tyrosine, or valine. After 3 h incubation at 37 °C, most CrH was observed in the sample with proline amide (125 μM), followed by the sample with valine (69 μM, Figure 3A). No significant CrH formation was observed with arginine, serine or threonine amides, although evidence for putative imidazolidin‐4‐ones was observed in all samples (note full characterization was hindered by signal overlap, Figure S10, Supporting Information). After 24 h, CrH was observed in all samples, with the most significant quantities being observed with proline and valine amides (196 and 213 μM respectively, Figure 3D). Therefore, it appears that proline amide is the most proficient catalyst for CrH formation from AcH over shorter reaction times.

Finally, we conducted experiments with a dimer of histones H2A and H2B, which contains an N‐terminal proline residue on H2B. Histones are key components of chromatin and are therefore positioned close to DNA; however, they are not reported to act as catalysts in cells. Incubation of the H2A:H2B dimer (430 nM) with AcH or propionaldehyde (20 mM, note H2A acts to solubilize H2B) did reveal formation of CrH and 2‐methylpent‐2‐enal respectively; however, control experiments also showed CrH/2‐methylpent‐2‐enal formation to a similar extent (Figure S11, Supporting Information). This observation is likely due to the presence of Tris buffer from the protein stock, which contains a primary amine. Therefore, it is unlikely that the H2B N‐terminal proline residue significantly catalyzes CrH/2‐methylpent‐2‐enal formation under the tested conditions. This observation is not surprising given the low concentration of H2A:H2B (430 nM).

## 3 Conclusion

Overall, the NMR analyses with proline amide reveal acceleration of CrH formation from AcH. This acceleration was more significant with proline amide than with other amino acid amides, which suggests that proline amide and its derivatives can catalyze CrH formation in cells. While our studies with recombinantly produced H2A:H2B suggest that such processes are likely to be low‐level in cells and are potentially out‐competed by other amine‐catalyzed pathways, the combined results imply that such reactions should dominate where localized proline amide and/or N‐terminal proline‐containing protein concentrations are increased. This might be the case on chromatin, where CrH‐derived DNA adducts occur after ethanol exposure and where an N‐terminal proline‐containing protein (H2B) is bound to DNA. The work also further highlights the complexity of disease‐relevant aldehyde (bio)chemistry in cells.

## Conflict of Interest

The authors declare no conflict of interest.

## Supporting information

Supplementary Material

## Data Availability

The data that support the findings of this study are available from the corresponding author upon reasonable request.
