# Supplementary material for: Proline Amide Catalyzes Formation of Toxic Crotonaldehyde from Acetaldehyde Under Physiologically Relevant Conditions
Source: Chembiochem. 2025 May 21;26(12):e202500138. doi: 10.1002/cbic.202500138 (PMC12177689; doi:10.1002/cbic.202500138)
Supplement: Supplementary file 1 — Supplementary Material [file CBIC-26-e202500138-s001.pdf]

Supporting Information

Proline amide catalyses formation of toxic crotonaldehyde from acetaldehyde under physiologically relevant conditions

Liam A. Thomas,[a] Vicki L. Emms,[a] Dipti Vashi,[b] Louise Fairall,[b] John W. R. Schwabe,[b] and Richard J. Hopkinson*[a]

[a] Dr L. A. Thomas, Dr V. L. Emms, Dr R. J. Hopkinson
Institute for Structural and Chemical Biology and School of Chemistry
University of Leicester
Henry Wellcome Building, Lancaster Road, Leicester, LE1 7RH, UK
E-mail: richard.hopkinson@leicester.ac.uk

[b] Mrs D. Vashi, Dr L. Fairall, Prof J. W. R. Schwabe
Institute for Structural and Chemical Biology and Department of Molecular and Cell Biology
University of Leicester
Henry Wellcome Building, Lancaster Road, Leicester, LE1 7RH, UK

Reagents

Reagents used in this work were purchased from the following suppliers:

Acetaldehyde (AcH, Acros Organic), propionaldehyde (Acros Organic), crotonaldehyde (CrH, Aldrich), paraformaldehyde (Sigma Aldrich), cinnamaldehyde (Fischer Scientific), alanine amide (Fluorochem), arginine amide (Fluorochem), glycine amide (Activate Scientific), histidine amide (Fluorochem), isoleucine amide (Fluorochem), leucine amide (Fluorochem), methionine amide (Fluorochem), proline amide (Fluorochem), phenylalanine amide (Fluorochem), serine amide (Fluorochem), threonine amide (Fluorochem), tryptophan amide (Fluorochem), tyrosine amide (Activate Scientific), valine amide (Fluorochem), 2'-deoxyguanosine monohydrate (Activate Scientific), 3-(trimethylsilyl)-2,2,3,3-tetradeuteropropionic acid (TSP, Thermo Fischer), D_2O (Apollo Scientific), Tris hydrochloride

(Apollo Scientific), monosodium phosphate monohydrate (Santa Cruz), disodium phosphate (MP Biomedicals), Rosetta2 (DE3) pLysS cells (Novagen), isopropyl ß-D-1-thiogalactopyranoside (IPTG, Melford), Complete EDTA-free protease inhibitor (Roche), Triton X-100 (Sigma), guanidine hydrochloride (Melford), urea (Melford), dithiothreitol (DTT, Melford), sodium chloride (Fisher), EDTA (Fisher), TCEP (Melford).

Production of buffers

0.1 M sodium phosphate buffer was prepared by dissolving 3.1 g of monosodium phosphate monohydrate and 10.9 g of anhydrous disodium phosphate into 1 L of 18.2 MΩ·cm MiliQ water and allowed to stir for 5 minutes until completely dissolved. Following dissolution, the pH was adjusted to pH 5/7.4/10 accordingly using 1-4 M NaOH or HCl. Buffers was stored at 4 °C between uses.

Production of formaldehyde (HCHO) stock solution

Paraformaldehyde (300 mg) was added to 10 mL of 18.2 MΩ·cm MiliQ water in a Schlenk tube fitted with a young's valve and sealed. The mixture was heated to 100 °C and stirred until no visible solid remained. The stock solution was stored at room temperature in the tube until required.

NMR Experiments

All NMR analyses were conducted on a Bruker 500 MHz spectrometer equipped with a 5 mm BBO probe and TopSpin 4.0.1 software. Data processing used Topspin 4.1.4 software. Samples with amino acid amides were prepared in microcentrifuges tubes (600 µL total volume) and contained the amino acid amide (12 µL of 100 mM stocks in DMSO-D_6), aldehyde (12 µL of 1 M stocks in H_2O, TSP (5 µL of a 58 mM stock in H_2O), D_2O (150 µL) and 100 mM sodium phosphate buffer in H_2O pre-buffered to pH 5, pH 7.4 or pH 10 (421 µL). Samples with H2A:H2B dimer contained the dimer (60 µL of 4.3 µM stock in 25 mM Tris, 2 M NaCl, 1 mM EDTA, 5 mM TCEP in H_2O pH 7.5), aldehyde (12 µL of 1 M stocks in H_2O), TSP (5 µL of a 58

mM stock in H_2O), D_2O (150 µL) and 100 mM sodium phosphate buffer in H_2O pH 7.4 (373 µL). After mixing, samples were transferred to Norell NMR tubes (5 mm) for NMR analysis.

Expression of histones H2A and H2B

H2A and H2B DNA cloned into pET3a plasmids were transformed separately into Rosetta2 (DE3) pLysS cells. Cultures of the transformed cells (750 mL total volume) were then grown to 0.6 A600 at 37°C before being induced with IPTG (0.4 mM). The cultures were then grown for a further 2 hours at 37°C before pelleting and freezing.

Formation and purification of the H2A:H2B dimer

The cell pellets containing histones H2A and H2B were separately defrosted and resuspended in histone wash buffer (50 mM Tris.HCl pH 7.5, 100 mM NaCl, 5 mM DTT and Complete EDTA-free protease inhibitor, 30 mL total volume). The cells were then sonicated and the insoluble fractions were pelleted by centrifugation at 4°C at 30000 g for 15 minutes. Each pellet was resuspended and re-pelleted twice with histone wash buffer with added 2% Triton X-100, and then three times with histone wash buffer only. Each pellet was then solubilised in DMSO (0.5 mL) by incubating on a roller at room temperature for 30 minutes. Unfolding buffer (7 M guanidine hydrochloride, 20 mM Tris.HCl, 10 mM DTT pH 7.5, 5 mL total volume) was then added, and each solution was incubated for an additional hour. The guanidine was diluted with 7 M urea, 50 mM Tris.HCl pH 7.5 and 1 mM EDTA (70 mL) and the samples were centrifuged at 35000 g for 20 minutes. Each histone solution was then filtered through a 0.22 µm filter prior to loading on a 5 mL HiTrap Q HP (Cytiva). The flow through from the HiTrap Q HP was then loaded on a 5 mL HiTrap SP HP (Cytiva). The histones were eluted using a gradient of 0-2 M NaCl. Histone-containing fractions were then dialysed into aqueous 0.1% acetic acid solution containing 5 mM DTT. Each histone solution was then aliquoted and freeze dried. Intact mass spectrometry was performed to confirm that the histones were full length (Figure S12).

The H2A:H2B heterodimer was prepared by combining equimolar amounts of H2A and H2B dissolved in unfolding buffer (7 M guanidine hydrochloride, 20 mM Tris pH 7.5 and 10 mM DTT). The sample was then dialysed against high salt buffer (25 mM Tris pH 7.5, 2.0 M NaCl, 1 mM EDTA and 5 mM TCEP) and then purified by size exclusion chromatography using a 10/300 Superdex S200 column (Cytiva) in the same buffer (Figure S13).

Supporting Figures

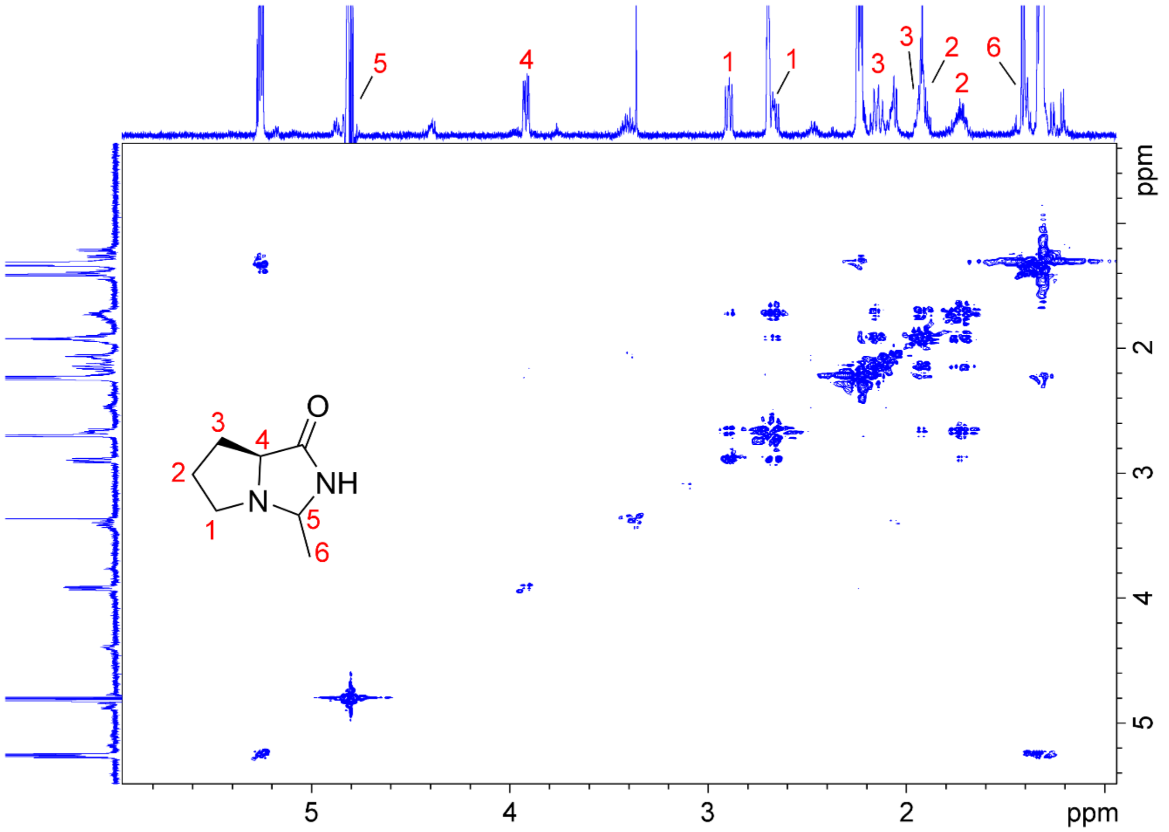

Figure S1. ^{1}H-^{1}H-COSY spectrum of a mixture of proline amide and AcH after 72 hours at 25 °C.

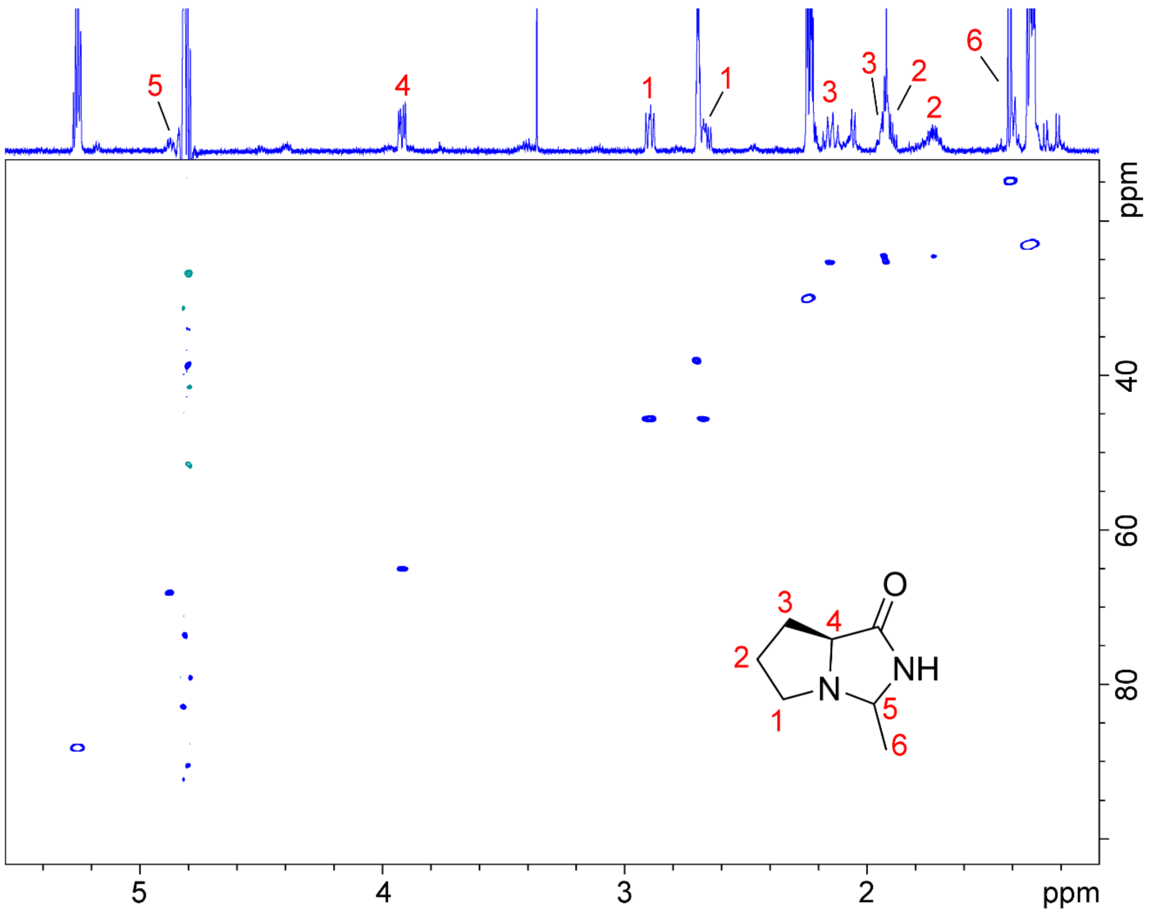

Figure S2. ^{1}H-^{13}C-HSQC spectrum of a mixture of proline amide and AcH after 72 hours at 25 °C.

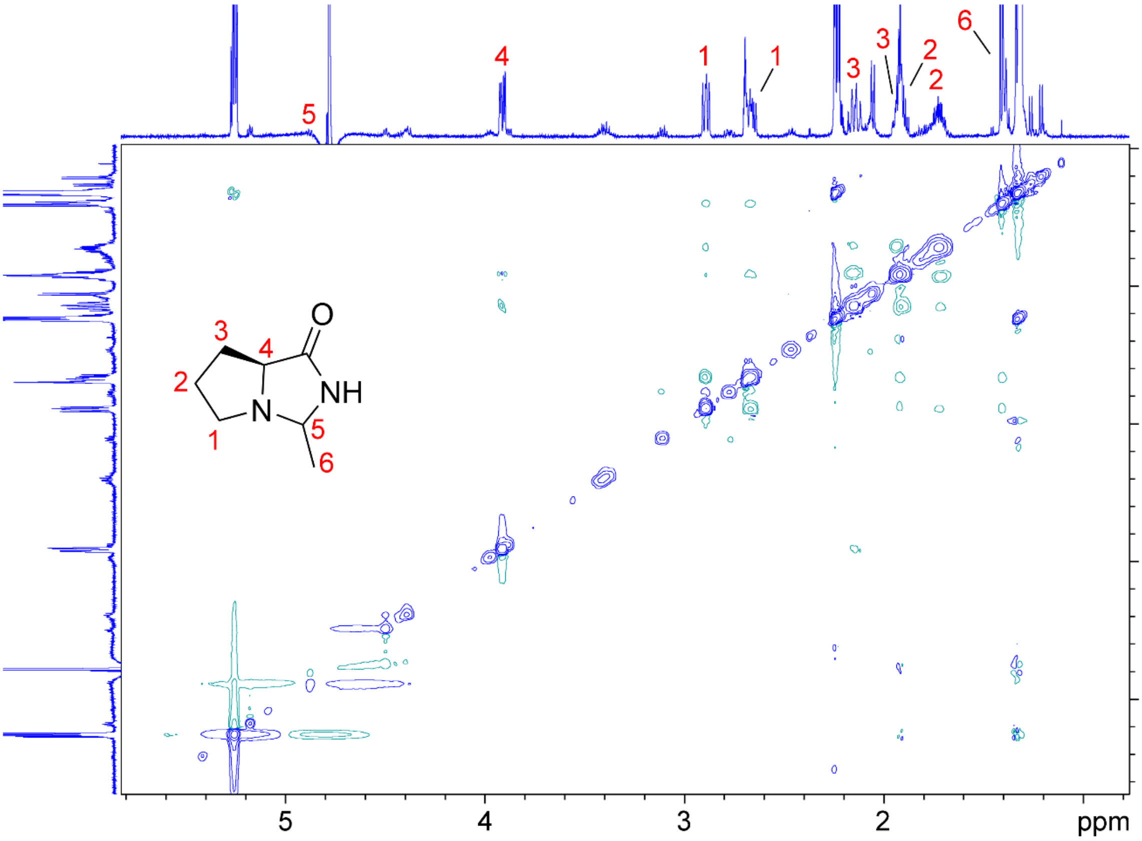

Figure S3. ^{1}H-^{1}H-NOESY spectrum of a mixture of proline amide and AcH after 90 hours at 25 °C.

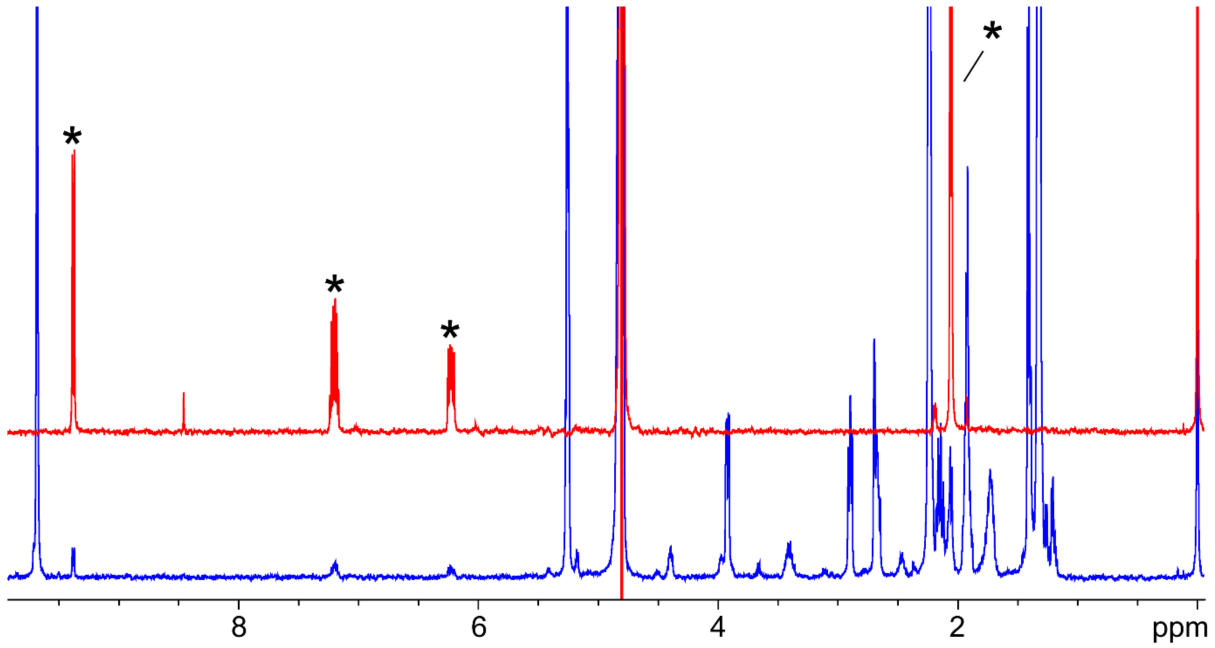

Figure S4. ^{1}H NMR spectra showing a mixture of proline amide and AcH after 72 hours at 25 °C (blue) and a sample of authentic CrH (red). ^{1}H resonances corresponding to CrH are highlighted (asterisks).

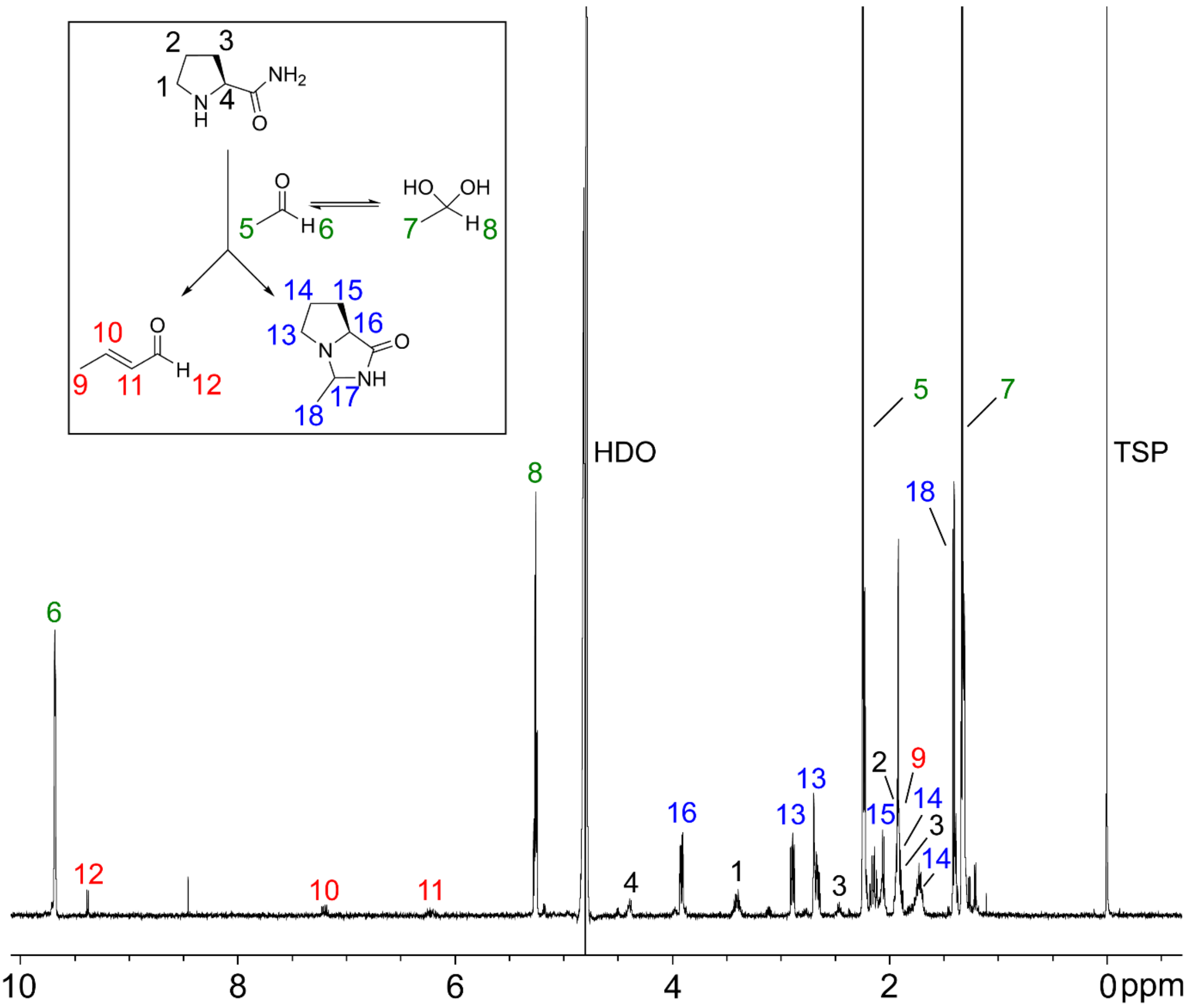

Figure S5. ^{1}H NMR spectrum of a mixture of proline amide and AcH after 24 hours at 37 °C. ^{1}H resonances corresponding to AcH, proline amide, CrH and MPI are highlighted.

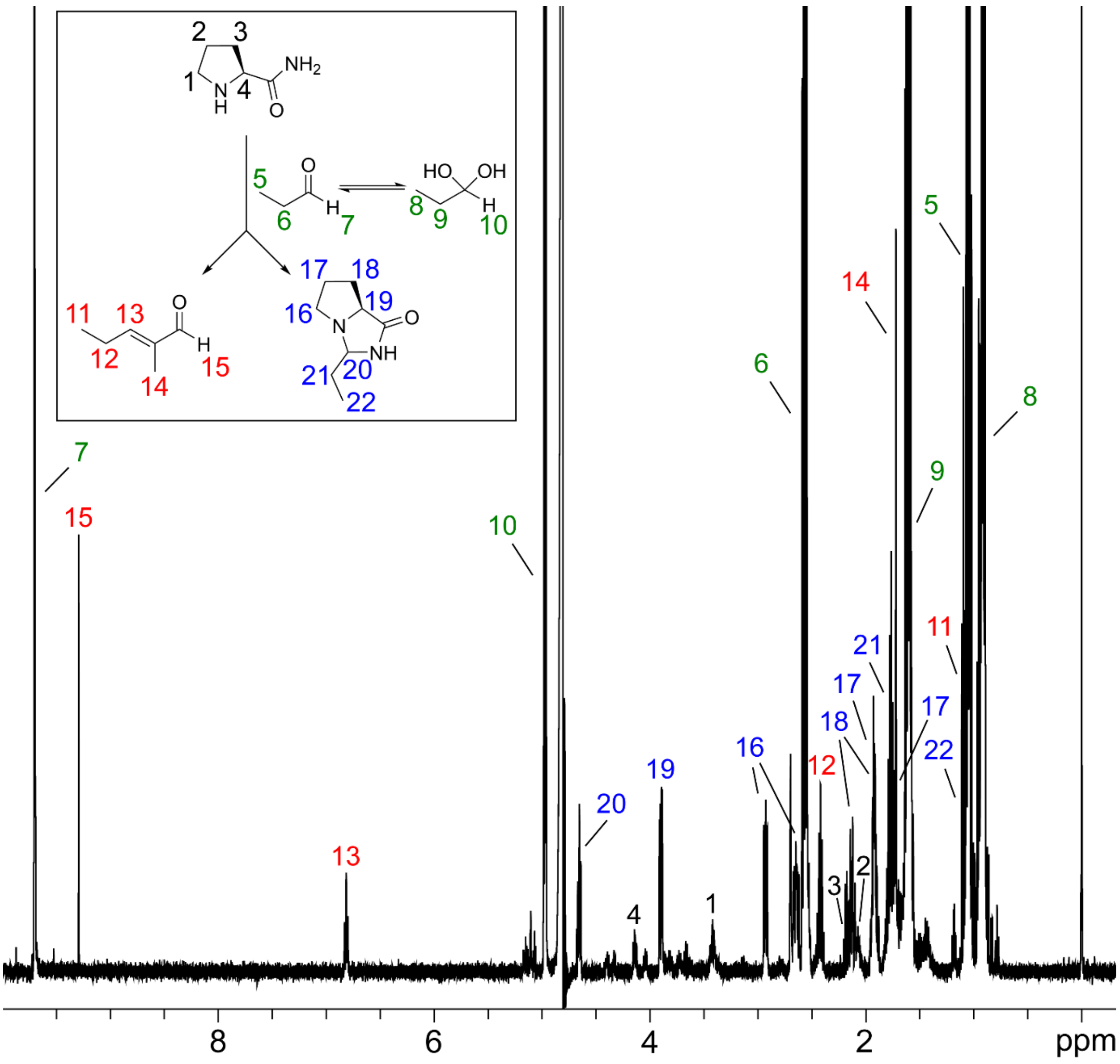

Figure S6. ^{1}H NMR spectrum of a mixture of proline amide and propionaldehyde after 60 hours at 25 °C. ^{1}H resonances corresponding to propionaldehyde, proline amide, (*E*)-2-methylpent-2-enal and the imidazolidin-4-one are highlighted.

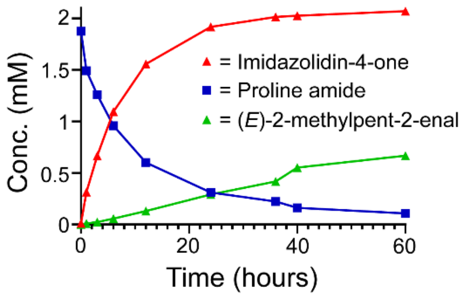

Figure S7. Graph showing time-dependent formation of (*E*)-2-methylpent-2-enal and an imidazolidin-4-one in a mixture of proline amide and propionaldehyde at pH 7.5 and 25 °C.

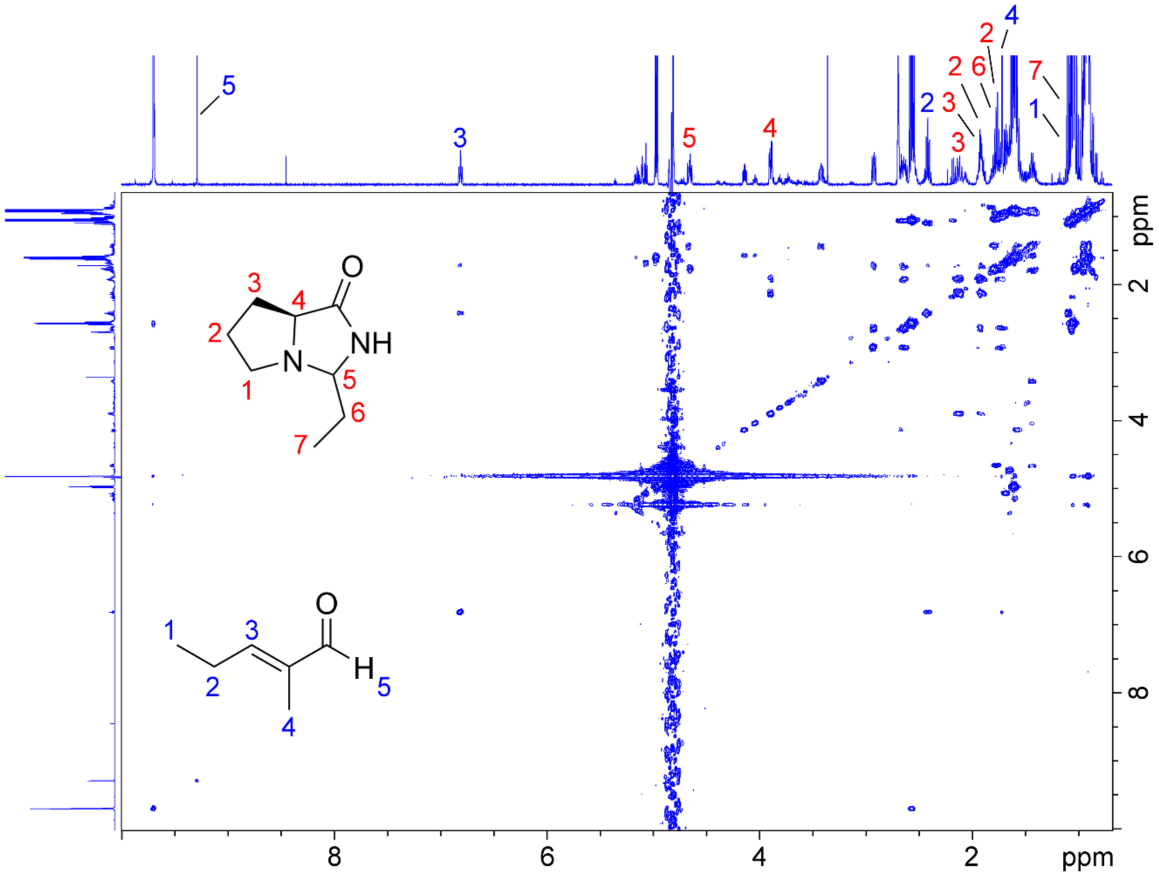

Figure S8. ^{1}H-^{1}H-COSY spectrum of a mixture of proline amide and propionaldehyde after 24 hours at 25 °C.

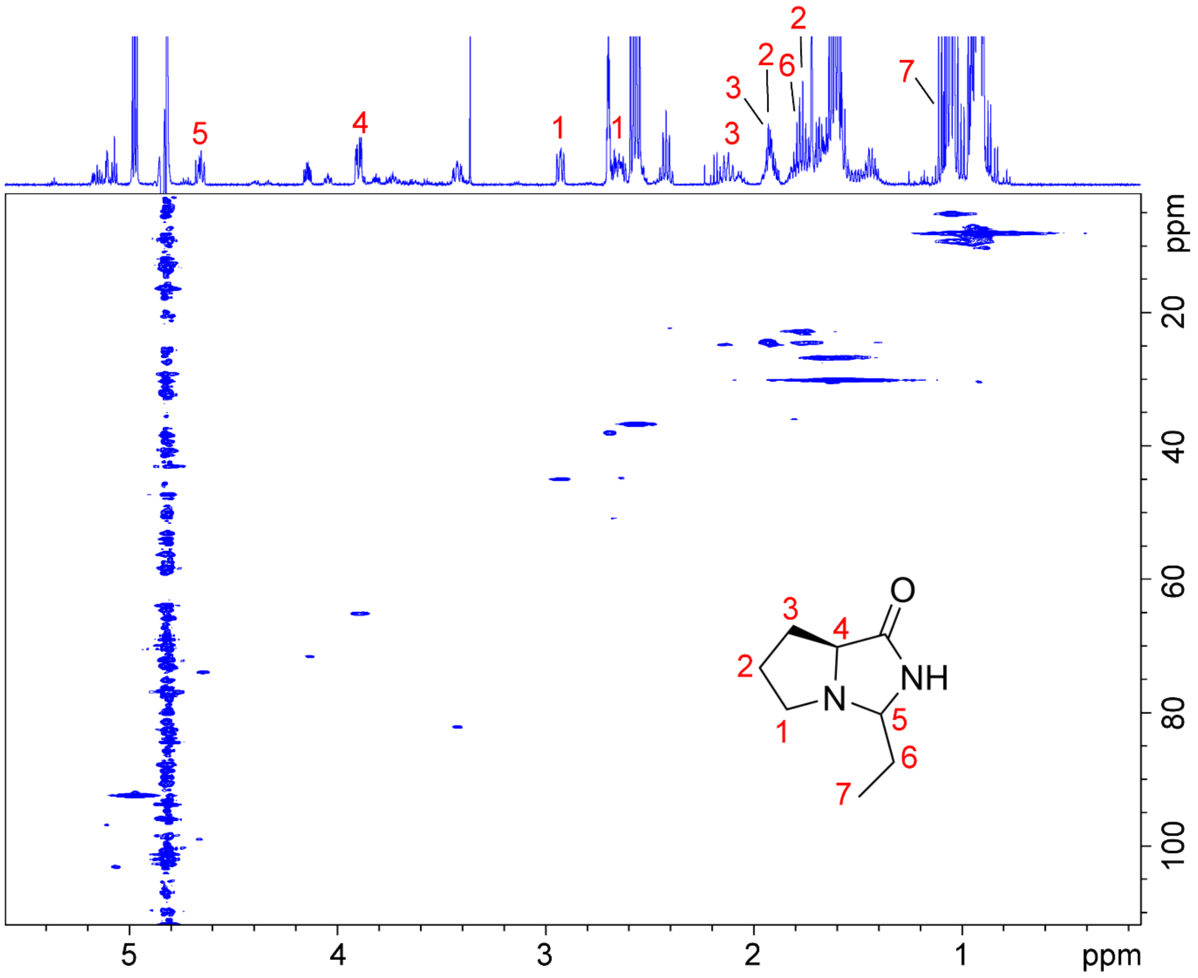

Figure S9. ^{1}H-^{13}C-HSQC spectrum of a mixture of proline amide and propionaldehyde after 24 hours at 25 °C.

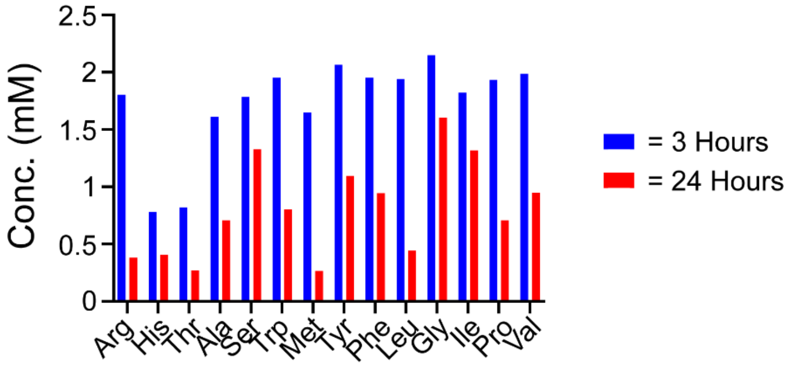

Figure S10. Bar graph showing concentrations of residual amino acid amides in mixtures of AcH and amino acid amides after 3 hours incubation at 37 °C. Initial amino acid amide concentrations were 2 mM.

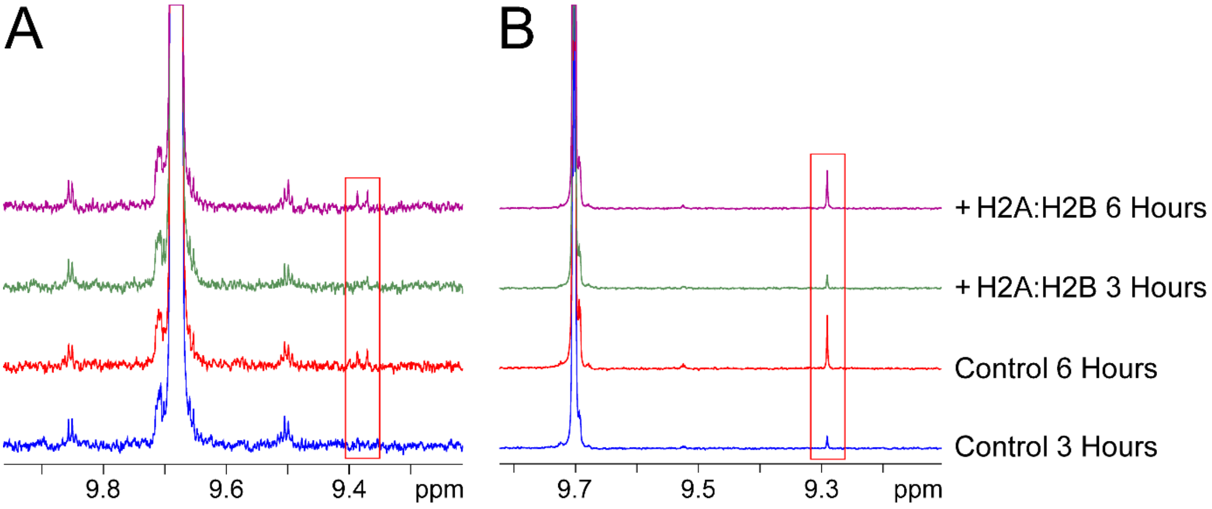

Figure S11. (A) ^{1}H NMR spectra showing time-dependent formation of CrH from AcH (20 mM) and the absence and presence of H2A:H2B dimer (430 nM). The ^{1}H resonance corresponding to CrH is highlighted in the red box. (B) ^{1}H NMR spectra showing time-dependent formation of 2-methylpent-2-enal from propionaldehyde (20 mM) and the absence and presence of H2A:H2B dimer (430 nM). The ^{1}H resonance corresponding to (*E*)-2-methylpent-2-enal is highlighted in the red box.

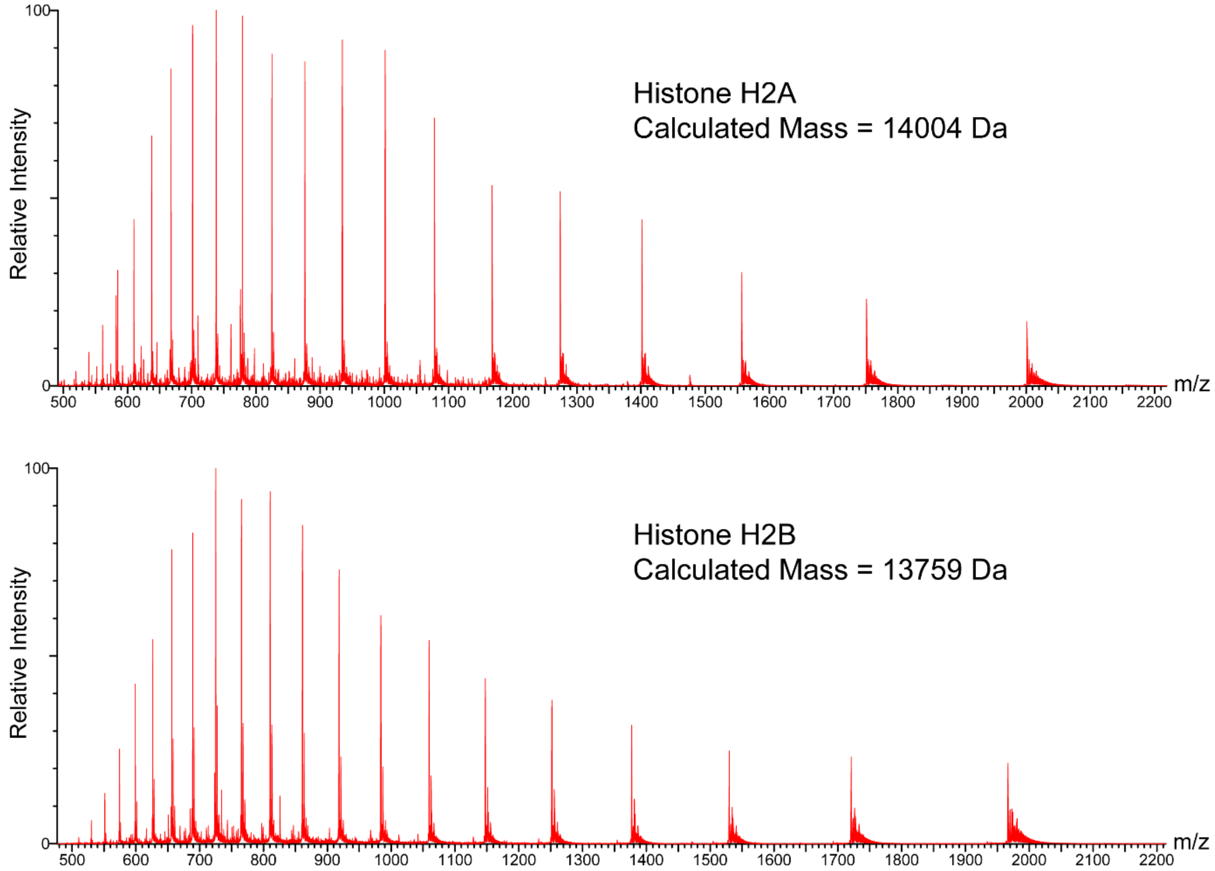

Figure S12. Mass spectra of recombinant histones H2A and H2B produced in Rosetta2 (DE3) pLysS cells.

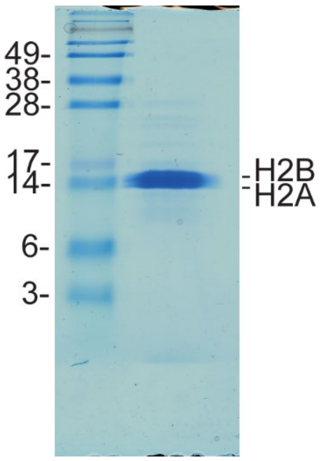

Figure S13. SDS-PAGE gel of the H2A:H2B heterodimer (denaturing conditions). A band corresponding to histones H2A and H2B is observed.
